# Localized tracheobronchial amyloidosis: A rare case presentation and tailored management approaches

**DOI:** 10.1002/rcr2.820

**Published:** 2021-08-10

**Authors:** Jonathan Arulanantham, Charlotte Officer, Chelsie O'Connor, Tina Baillie, Simon Bass, Jonathan P. Williamson, Alan Carew

**Affiliations:** ^1^ Faculty of Medicine Health and Human Sciences Macquarie University Macquarie Park New South Wales Australia; ^2^ GenesisCare, Radiation Oncology Macquarie University Hospital, Macquarie University Macquarie Park New South Wales Australia; ^3^ Anatomical Pathology Douglass Hanly Moir Macquarie Park New South Wales Australia; ^4^ Medical Services Group South East Regional Hospital Bega New South Wales Australia; ^5^ South Western Sydney Clinical School, Liverpool Hospital The University of New South Wales Sydney New South Wales Australia; ^6^ MQ Health Respiratory and Sleep Macquarie University Hospital Macquarie Park New South Wales Australia; ^7^ School of Medicine University of Queensland Brisbane Queensland Australia

**Keywords:** amyloidosis, bronchoscopy and interventional techniques, interventional pulmonology, obstructive lung disease, respiratory structure and function

## Abstract

Localized tracheobronchial amyloidosis (TBA) is a rare manifestation of pulmonary amyloid disease, and can result in central airway obstruction. The nature of presentation is variable and there may be a delayed diagnosis. TBA has a variable prognosis and the most commonly used strategy for management is airway recanalization. Here, we describe the tailored management approach for a 64‐year‐old Caucasian female presenting with localized TBA of the left main bronchus. Pulmonary function testing, computed tomography and positron emission tomography results are detailed. Rigid bronchoscopy was utilized for diagnostic and therapeutic intervention, which involved debulking and stent insertion. Amyloid deposition and localized inflammation were identified through histopathology. Focal external beam radiation therapy was administered following multidisciplinary discussion and review of the literature, with no evidence of active disease at 6 months follow‐up.

## Graphical abstract

Localized tracheobronchial amyloidosis (TBA) is a rare manifestation of pulmonary amyloidosis, resulting from the pathological deposition of amyloid protein within the central airway wall. We present the initial management and subsequent outcomes of a patient with localized TBA, presenting with central airway obstruction.

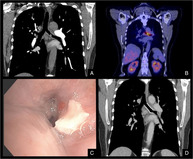

## INTRODUCTION

Localized tracheobronchial amyloidosis (TBA) is a rare manifestation of pulmonary amyloidosis, resulting from the pathological deposition of amyloid protein within the central airway wall.^1^, ^2^, ^3^ Due to structural changes and luminal narrowing, TBA contributes to progressive respiratory symptoms, which are frequently non‐specific and may mimic other common obstructive respiratory diseases.^1^, ^2^, ^3^ Imaging findings are varied and often non‐specific, contributing to diagnostic delay.^2^ TBA has a variable prognosis and management entails treating an underlying condition if present, decreasing the amount of amyloid present and providing relief of airway obstruction.^1^, ^2^ We present the initial management and subsequent outcomes of a patient with localized TBA, presenting with central airway obstruction.

## CASE REPORT

A 64‐year‐old Caucasian female was referred to our institution with a month‐long history of progressive dyspnoea, productive cough, wheeze and intermittent stridor. She was a non‐smoker with a background history of hypertension and hepatic steatosis. She had presented several times to medical attention and was prescribed empiric antibiotics and corticosteroid therapy for suspected asthma or bronchitis, without improvement.

Physical examination of the chest revealed inspiratory stridor and a fixed monophonic wheeze across the left lung field. Examination of the right chest was normal. The remainder of her examination was unremarkable, with no evidence of skin thickening or bruising, macroglossia, enlargement of the parotid or submandibular glands or periorbital purpura.

**FIGURE 1:**
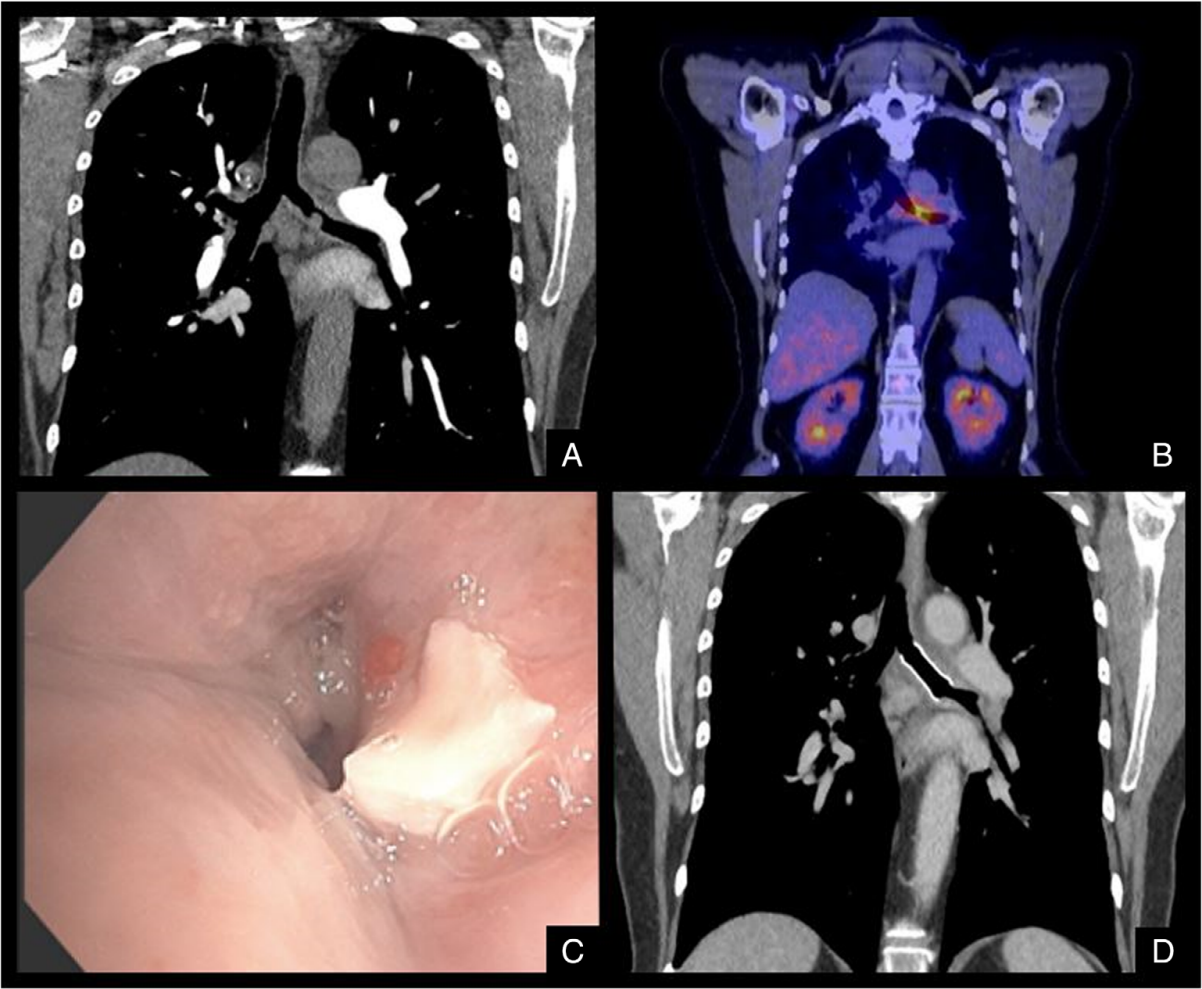
(A) Mediastinal view of computed tomography (CT) pulmonary angiogram showing mass in the left main bronchus (LMB). (B) Positron emission tomography/CT scan showing fluorodeoxyglucose uptake at the LMB and subcarinal, mediastinal and hilar lymph nodes. (C) Bronchoscopic image identifying obstructed LMB from the carina. (D) Coronal CT chest showing a metallic stent in the LMB

Chest radiography was unremarkable. Progressive symptoms and development of haemoptysis prompted computed tomography pulmonary angiography to exclude pulmonary embolism. A mass was detected in the left main bronchus (LMB) (Figure 1A). Bronchoscopy for tissue sampling of the lesion was performed, identifying amyloid deposits on histopathology. Positron emission tomography demonstrated moderate fluorodeoxyglucose (FDG) avidity at the LMB lesion site (maximum standardised uptake value [SUV_max_] = 6) and mild FDG avidity in the subcarinal lymph node, with no apparent evidence of disease activity in other sites (Figure 1B).

Transthoracic echocardiography suggested mild diastolic dysfunction. Respiratory function tests showed airflow limitation, with normal lung volumes and carbon monoxide diffusion capacity (forced expiratory volume in 1 s/forced vital capacity [FEV_1_/FVC] = 1.64/3.10 L = 53%). A positive bronchodilator response was noted and interpreted as the result of improved spirometry technique with repeated manoeuvres (% change in FEV_1_ = 18% and 290 ml; % change in FVC = 12% and 380 ml). Inflammatory markers were elevated (erythrocyte sedimentation rate = 50 mm/h; C‐reactive protein = 6.3 mg/L).

The patient underwent rigid and flexible bronchoscopy which demonstrated friable, oedematous and cobblestoned mucosa in the LMB, with a pearlescent irregular mass protruding from the anterior bronchial wall (Figure 1C). There was partial obstruction of both upper and lower lobe bronchi. The lesion was biopsied and debulked with a 2.4‐mm cryoprobe, followed by controlled radial expansion balloon dilatation. Extensive malacia of the LMB was evident with complete airway collapse on expiration, so a 12 × 30 mm fully covered, self‐expanding metallic stent was inserted (Figure 1D).

**FIGURE 2:**
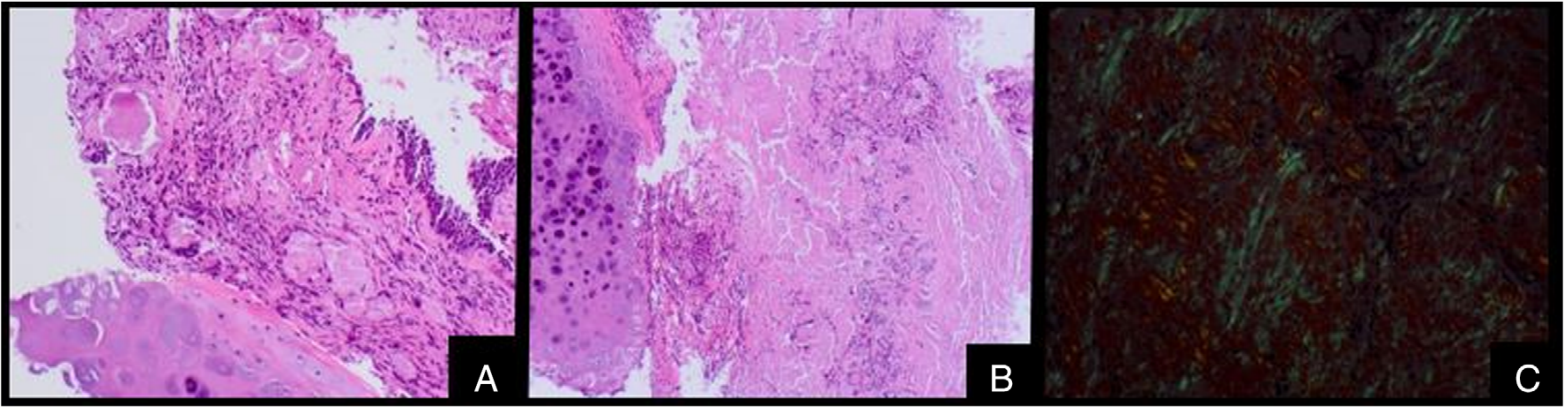
Histology. (A) Haematoxylin and eosin (H&E) image showing a globular pattern of deposition of amorphous eosinophilic material around native mucus gland. (B) H&E images showing a more diffuse pattern of deposition. (C) Apple‐green birefringence on Congo red staining under polarized light microscopy

Histopathology confirmed hyperplastic ciliated bronchial epithelium with mucosal erosions, fibrinous exudate and fibroinflammatory changes, with polypoid ingrowth of inflamed granulation tissue (Figure 2A,B). There was diffuse thickening of the bronchial wall by submucosal extracellular homogenous eosinophilic material, with associated focal foreign body giant cell reactions, surrounding seromucous glands and cartilage plates. Congo red staining confirmed congophilia with apple‐green birefringence under polarized light, confirming the amyloid deposition (Figure 2C). Endobronchial ultrasound‐guided needle aspirates of the mildly FDG‐avid subcarinal node reflected reactive changes.

The patient received external beam radiation therapy at a dose of 20 Gy in 10 fractions following multidisciplinary expert advice and recommendations from previously published case series data.^4^ This dosing schedule provides the option of repeat treatment, if required, in future for disease recurrence.^4^ The covered metal airway stent was removed after 6 weeks, with minimal residual airway obstruction. However, she was symptomatic of localized bronchomalacia with mucous inspissation, necessitating the insertion of a silicone airway stent, 6 months following radiation therapy.

## DISCUSSION

Respiratory amyloidosis can manifest in three ways: nodular pulmonary amyloidosis, diffuse parenchymal amyloidosis and localized TBA.^1^ Nodular pulmonary amyloidosis is notably benign, describing localized nodular amyloid deposits, whilst diffuse parenchymal amyloidosis is often associated with systemic disease.^2^, ^3^, ^4^ Generally an acquired disease, TBA manifests as submucosal plaques primarily limited to the central airways, with rare multifocal manifestations.^1^, ^2^, ^3^, ^5^

Symptoms associated with TBA result from focal airway obstruction and include dyspnoea, cough and sputum production, haemoptysis and wheeze. These are non‐specific in nature, which contributes to the delay of a definitive diagnosis.^1^, ^2^, ^5^ The disease location within the airway determines the acuity of the presentation, with tracheal TBA potentially leading to critical airway obstruction. More distal bronchial TBA may lead to lobar atelectasis, pneumonia or bronchiectasis.^1^, ^2^ Mortality of 30% after 7‐ to 12‐year follow‐up of TBA patients has been documented.^5^

The most common therapeutic strategy described for localized TBA is bronchoscopic airway debulking using a combination of airway ablative therapies and/or tracheobronchial stenting.^1^, ^2^ Pharmacological measures, such as corticosteroids and colchicine, may be beneficial in systemic amyloidosis, but are not recommended as primary intervention for localized TBA.^1^, ^5^ External beam radiation therapy can be used for localized TBA, by either directly targeting the amyloid deposition or inducing an inflammatory response.^2^, ^4^

Multi‐modal therapeutic strategies combining airway recanalization and radiotherapy have been suggested in limited reports.^2^ The negative short‐term effects include radiation oesophagitis, pneumonitis and bleeding.^1^, ^2^, ^4^ However, long‐term local disease control has been documented in limited studies.^1^, ^2^, ^4^ At 6 months, our patient has achieved disease control and airway patency, and we plan 6‐monthly clinical, spirometry and bronchoscopic surveillance. Repeat external beam radiotherapy is also possible, if her disease recurs.

## CONFLICT OF INTEREST

None declared.

## ETHICS STATEMENT

Appropriate written informed consent was obtained for publication of this case report and accompanying images.

## AUTHOR CONTRIBUTIONS

A/Prof Jonathan P. Williamson and Dr Alan Carew were treating clinicians and main supervisors. Dr Tina Baillie, Dr Chelsie O'Connor and Dr Simon Bass were treating clinicians and were involved in the redaction of the manuscript.
